# Supplementary material for: svclassify: a method to establish benchmark structural variant calls
Source: BMC Genomics. 2016 Jan 16;17:64. doi: 10.1186/s12864-016-2366-2 (PMC4715349; doi:10.1186/s12864-016-2366-2)
Supplement: Additional file 5: Table S5. — Selected characteristics for hierarchical clustering and One-class models. (DOC 23 kb) [file 12864_2016_2366_MOESM5_ESM.docx]

**Supplementary table 5**: Selected characteristics for hierarchical clustering and One-class models.

For Illumina and moleculo datasets:

M_Cov, M_Cov_sd, M_Insert_sd, M_Dis_unmap_ratio, M_Dis_map_ratio, M_Soft_pro, M_Homvar_SV, M_Hetvar_SV

L_Insert_90_percentile, L_Dis_unmap_ratio, L_Dis_map_ratio, L_Soft_90_percentile, L_Cov, L_Insert_sd, L_Soft_pro

R_Insert_90_percentile, R_Dis_unmap_ratio, R_Dis_map_ratio, R_Soft_90_percentile, R_Cov

LM_Insert_90_percentile, LM_Dis_unmap_ratio, LM_Dis_map_ratio, LM_Soft_90_percentile, LM_Cov

RM_Insert_90_percentile, RM_Dis_unmap_ratio, RM_Dis_map_ratio, RM_Soft_90_percentile, RM_Cov

R_Insert_90_percentile, R_Dis_unmap_ratio, R_Dis_map_ratio, R_Soft_90_percentile, R_Cov

For PacBio datasets:

L_Diff, R_Diff, M_Cov, M_Del, M_Ins, M_Diff, M_Diff_sd

For hierarchical clustering only:

SV_size, M_Mapping_q, M_Sine_Line_Ltr_SV, M_GC_Content, and M_Simple_Low_Satellite_SV

- Note also that all of these except M_Mapping_q are the same for all of the datasets so only need to be included once for the joint dataset unsupervised analysis.
