# Supplementary material for: svclassify: a method to establish benchmark structural variant calls
Source: BMC Genomics. 2016 Jan 16;17:64. doi: 10.1186/s12864-016-2366-2 (PMC4715349; doi:10.1186/s12864-016-2366-2)
Supplement: Additional file 6: Figure S1. — Histogram of ρ score for 2306 random regions with same size distribution as Personalis SVs. (PDF 322 kb) [file 12864_2016_2366_MOESM6_ESM.pdf]

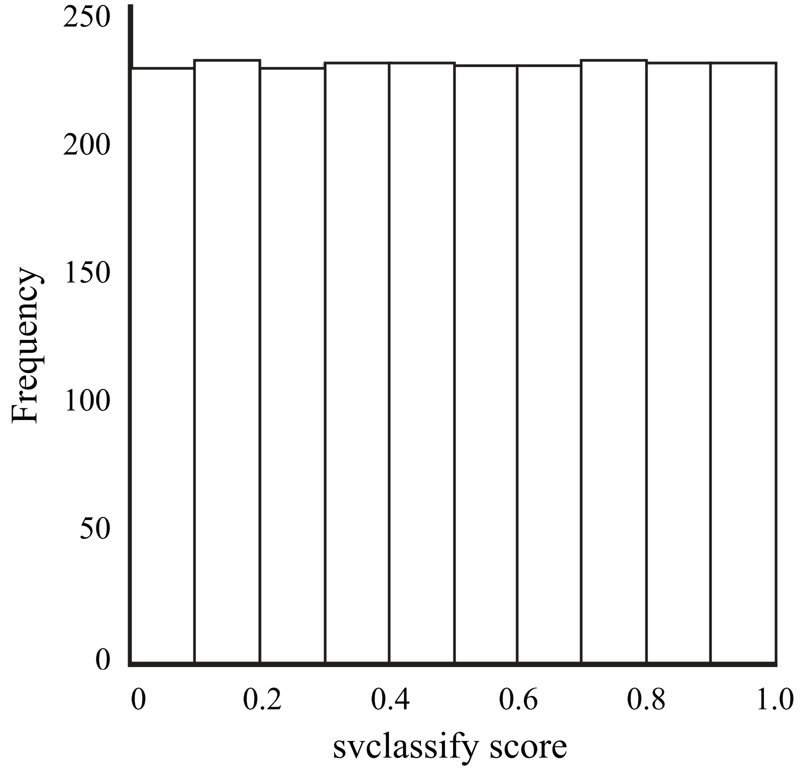

Supplementary figure 1: Histogram of ρ score for 2306 random regions with same size distribution as Personalis SVs.
