# Supplementary material for: svclassify: a method to establish benchmark structural variant calls
Source: BMC Genomics. 2016 Jan 16;17:64. doi: 10.1186/s12864-016-2366-2 (PMC4715349; doi:10.1186/s12864-016-2366-2)
Supplement: Additional file 7: Figure S2. — ROC curves for One-class classification using SVM, treating the 4000 Random regions as negatives and the Personalis or 1000 Genomes calls as positives. (A) ROC curves for one-class models for each dataset separately and for all combined for the Personalis validated deletion calls. (B) ROC curves for one-class models for each dataset separately and for all combined for the 1000 Genomes validated deletion calls. (C) ROC curves for one-class model requiring 1 or more, 2 or more, 3 or more, or all 4 technologies to have high classification scores for the Personalis validated deletion calls. (D) ROC curves for one-class model requiring 1 or more, 2 or more, 3 or more, or all 4 technologies to have high classification scores for the 1000 Genomes validated deletion calls. See original data at https://plot.ly/233/~parikhhm/, https://plot.ly/242/~parikhhm/, https://plot.ly/250/~parikhhm/, and https://plot.ly/258/~parikhhm/. (PDF 579 kb) [file 12864_2016_2366_MOESM7_ESM.pdf]

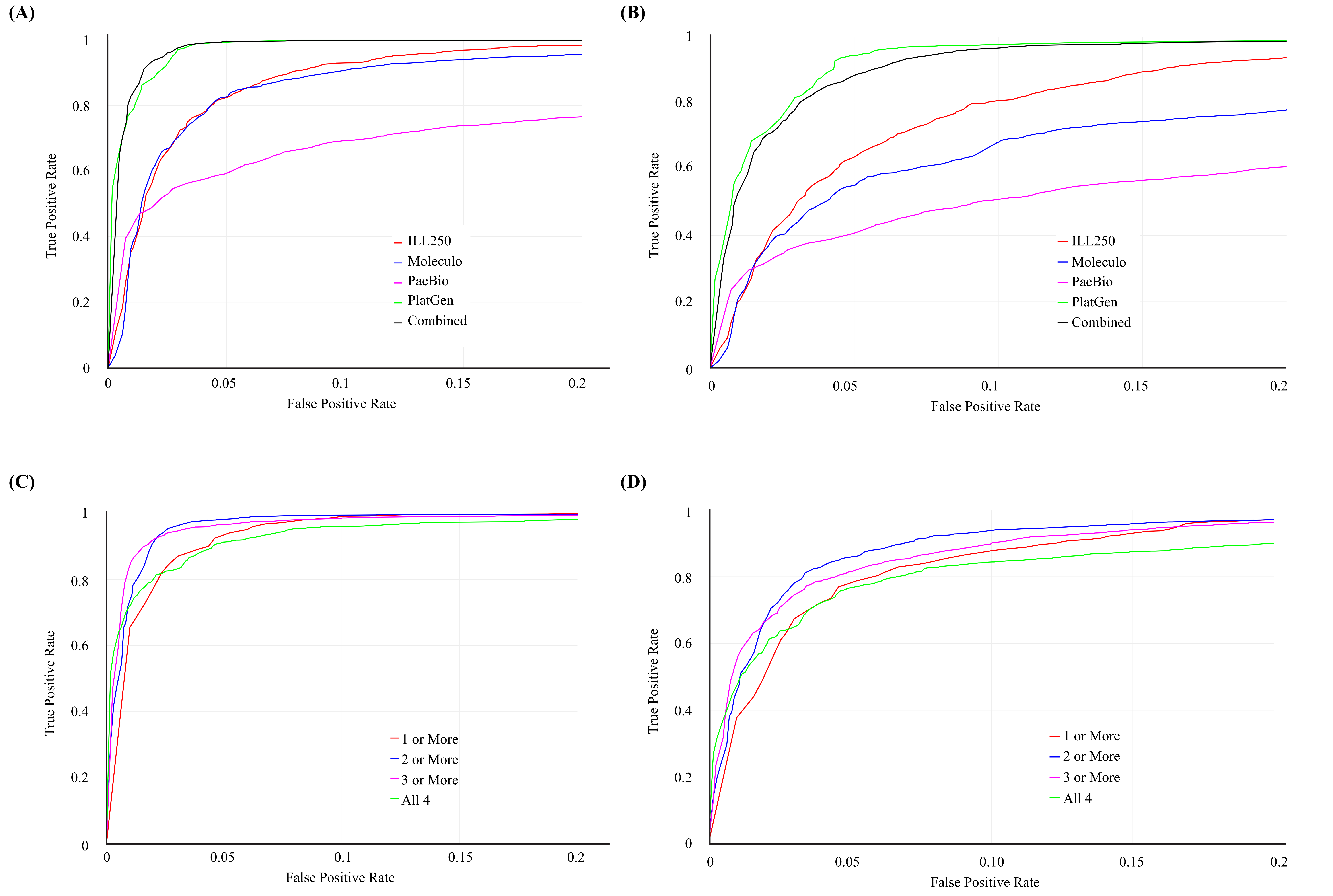

Supplementary figure 2: ROC curves for One-class classification using SVM, treating the 4000 Random regions as negatives and the Personalis or 1000 Genomes calls as positives. (A) ROC curves for one-class models for each dataset separately and for all combined for the Personalis validated deletion calls. (B) ROC curves for one-class models for each dataset separately and for all combined for the 1000 Genomes validated deletion calls. (C) ROC curves for one-class model requiring 1 or more, 2 or more, 3 or more, or all 4 technologies to have high classification scores for the Personalis validated deletion calls. (D) ROC curves for one-class model requiring 1 or more, 2 or more, 3 or more, or all 4 technologies to have high classification scores for the 1000 Genomes validated deletion calls. See original data at https://plot.ly/233/~parikhhm/, https://plot.ly/242/~parikhhm/, https://plot.ly/250/~parikhhm/, and https://plot.ly/258/~parikhhm/.
