# Supplementary material for: svclassify: a method to establish benchmark structural variant calls
Source: BMC Genomics. 2016 Jan 16;17:64. doi: 10.1186/s12864-016-2366-2 (PMC4715349; doi:10.1186/s12864-016-2366-2)
Supplement: Additional file 8: Figure S3. — ROC curves for One-class classification using SVM and L1 “3 or more” strategy, treating the 4000 random regions as training negatives, treating (A) the Personalis deletion calls and (B) the 1000 Genomes deletion calls as testing positives and treating the 2306 random regions as testing negatives. See original data at https://plot.ly/266/~parikhhm/, and https://plot.ly/274/~parikhhm/. (PDF 305 kb) [file 12864_2016_2366_MOESM8_ESM.pdf]

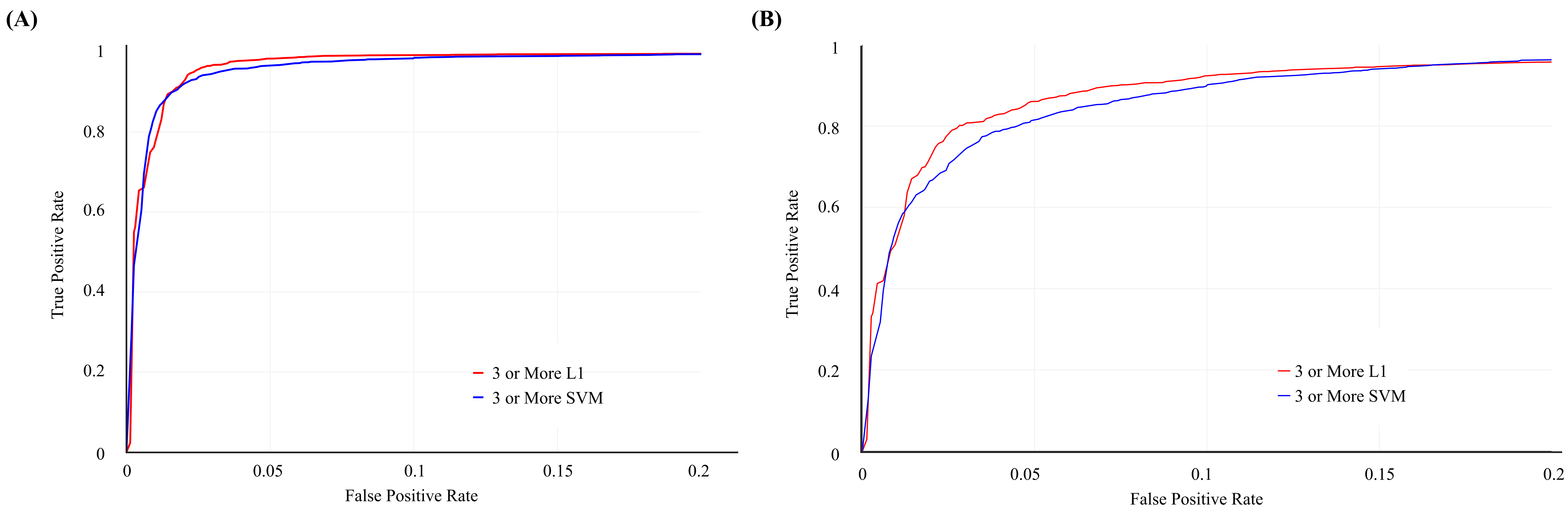

Supplementary figure 3: ROC curves for One-class classification using SVM and L1 "3 or more" strategy, treating the 4000 random regions as training negatives, treating (A) the Personalis deletion calls and (B) the 1000 Genomes deletion calls as testing positives and treating the 2306 random regions as testing negatives. See original data at https://plot.ly/266/~parikhhm/, and https://plot.ly/274/~parikhhm/.
