# Supplementary material for: svclassify: a method to establish benchmark structural variant calls
Source: BMC Genomics. 2016 Jan 16;17:64. doi: 10.1186/s12864-016-2366-2 (PMC4715349; doi:10.1186/s12864-016-2366-2)
Supplement: Additional file 9: Figure S4. — ROC curves for One-class classification using SVM, treating the 4000 random regions as negatives and the Spiral Genetics insertions calls as positives. (A) ROC curves for one-class models for each dataset separately and for all combined. (B) ROC curves for one-class model requiring 1 or more, 2 or more, 3 or more, or all 4 technologies to have high classification scores. See original data at https://plot.ly/~desuchen0929/391, and https://plot.ly/297/~parikhhm/. (PDF 351 kb) [file 12864_2016_2366_MOESM9_ESM.pdf]

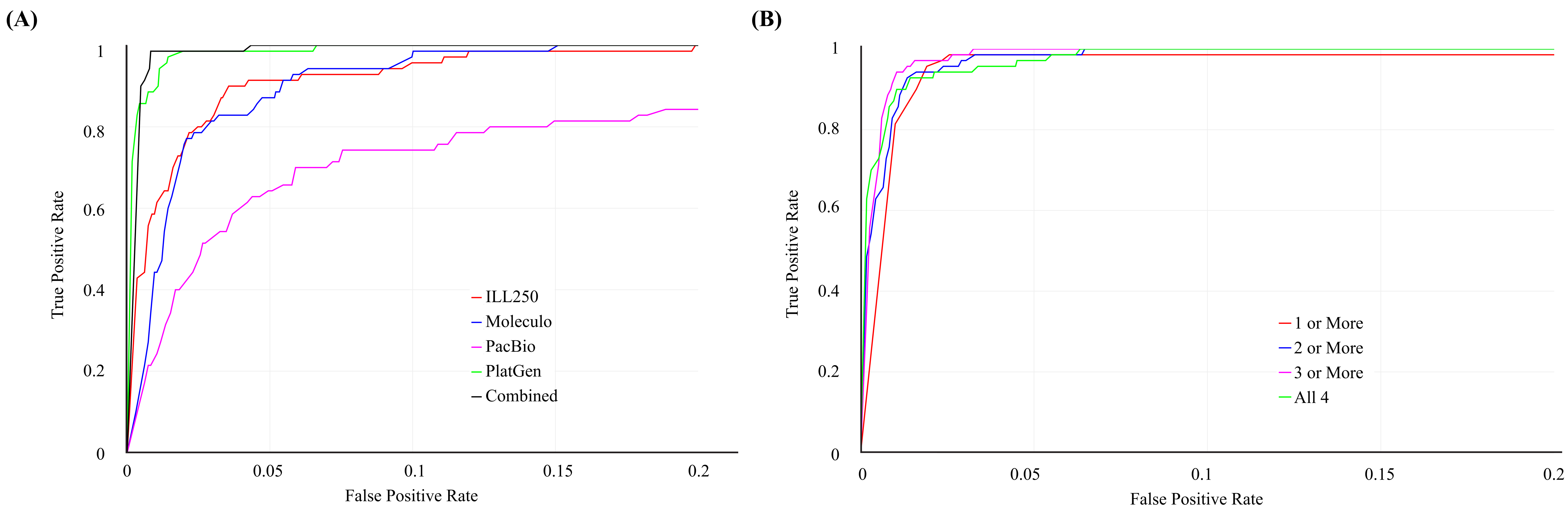

Supplementary figure 4: ROC curves for One-class classification using SVM, treating the 4000 random regions as negatives and the Spiral Genetics insertions calls as positives. (A) ROC curves for one-class models for each dataset separately and for all combined. (B) ROC curves for one-class model requiring 1 or more, 2 or more, 3 or more, or all 4 technologies to have high classification scores. See original data at https://plot.ly/~desuchen0929/391, and https://plot.ly/297/~parikhhm/.
