# Supplementary material for: svclassify: a method to establish benchmark structural variant calls
Source: BMC Genomics. 2016 Jan 16;17:64. doi: 10.1186/s12864-016-2366-2 (PMC4715349; doi:10.1186/s12864-016-2366-2)
Supplement: Additional file 11: Table S6. — Elements of concordance/discordance matrix of predictions on Personalis validated/assembled set by the one-class L1 classifier and one-class SVM with annotations of all technologies combined. (DOC 43 kb) [file 12864_2016_2366_MOESM11_ESM.docx]

**Supplementary table 6**: Elements of concordance/discordance matrix of predictions on Personalis validated/assembled set by the one-class L_1_ classifier and one-class SVM with annotations of all technologies combined.

| Ρ | 0.99 | 0.95 | 0.9 | 0.68 |
| --- | --- | --- | --- | --- |
| SVM(+), L_1_(+) | 1665 | 2291 | 2302 | 2306 |
| SVM(+), L_1_(-) | 458 | 4 | 1 | 0 |
| SVM(-), L_1_(+) | 10 | 5 | 0 | 0 |
| SVM(-), L_1_(-) | 173 | 6 | 3 | 0 |

Elements of concordance/ discordance matrix of predictions on Personalis validated/assembled set with ensemble classifiers (k=3) of one-class L_1_ classifier and one-class SVM.

| Ρ | 0.99 | 0.95 | 0.9 | 0.68 |
| --- | --- | --- | --- | --- |
| SVM(+), L_1_(+) | 1711 | 2232 | 2275 | 2296 |
| SVM(+), L_1_(-) | 341 | 12 | 4 | 3 |
| SVM(-), L_1_(+) | 45 | 35 | 11 | 1 |
| SVM(-), L_1_(-) | 209 | 27 | 16 | 6 |

Elements of concordance/Discordance matrix of predictions on 1000 Genomes set by the one-class L_1_ classifier and one-class SVM with annotations of all technologies combined.

| Ρ | 0.99 | 0.95 | 0.9 | 0.68 |
| --- | --- | --- | --- | --- |
| SVM(+), L_1_(+) | 1188 | 2373 | 2567 | 2654 |
| SVM(+), L_1_(-) | 598 | 100 | 51 | 7 |
| SVM(-), L_1_(+) | 100 | 43 | 7 | 8 |
| SVM(-), L_1_(-) | 799 | 169 | 60 | 16 |

Elements of concordance/ discordance matrix of predictions on 1000 Genomes set with ensemble classifiers (k=3) of one-class L_1_ classifier and one-class SVM.

| Ρ | 0.99 | 0.95 | 0.9 | 0.68 |
| --- | --- | --- | --- | --- |
| SVM(+), L_1_(+) | 1189 | 2161 | 2405 | 2598 |
| SVM(+), L_1_(-) | 463 | 93 | 69 | 41 |
| SVM(-), L_1_(+) | 176 | 150 | 75 | 16 |
| SVM(-), L_1_(-) | 857 | 281 | 136 | 30 |
