# Supplementary material for: svclassify: a method to establish benchmark structural variant calls
Source: BMC Genomics. 2016 Jan 16;17:64. doi: 10.1186/s12864-016-2366-2 (PMC4715349; doi:10.1186/s12864-016-2366-2)
Supplement: Additional file 20: Figure S6. — Three levels of validation with trio analysis. “Total Validated” gives the number of sites validated by that level or any level above. “Total not Validated” gives the number of sites not validated in that level or any level above. “Additionally Validated” gives the number of sites validated by that level, excluding sites validated in levels above. (PDF 296 kb) [file 12864_2016_2366_MOESM20_ESM.pdf]

| | Total Validated | Total not Validated | Additionally Validated |
|---|---|---|---|
| GIAB High-Confidence Deletions (HC) | 0 (0%) | 2348 (100%) | 0 (0%) |
| GIAB HC Validated by Parents (Level 1) | 2302 (98.0%) | 46 (2.0%) | 2302 (98.0%) |
| GIAB HC Validated by Child (Level 2) | 2306 (98.2%) | 42 (1.8%) | 4 (0.2%) |
| GIAB HC Validated by Child (Level 3) | 2342 (99.7%) | 6 (0.3%) | 36 (1.5%) |

**Supplementary figure 6**: Three levels of validation with trio analysis. "Total Validated" gives the number of sites validated by that level or any level above. "Total not Validated" gives the number of sites not validated in that level or any level above. "Additionally Validated" gives the number of sites validated by that level, excluding sites validated in levels above.
