# Supplementary material for: svclassify: a method to establish benchmark structural variant calls
Source: BMC Genomics. 2016 Jan 16;17:64. doi: 10.1186/s12864-016-2366-2 (PMC4715349; doi:10.1186/s12864-016-2366-2)
Supplement: Additional file 21: Figure S7. — ROC curves for One-class classification using the L1 Distance, treating the 2306 Random regions (size distribution matching to Personalis) as negatives and the Personalis as positives using the Platinum down-sampling 30x data. See original data at https://plot.ly/165/~parikhhm/. (PDF 169 kb) [file 12864_2016_2366_MOESM21_ESM.pdf]

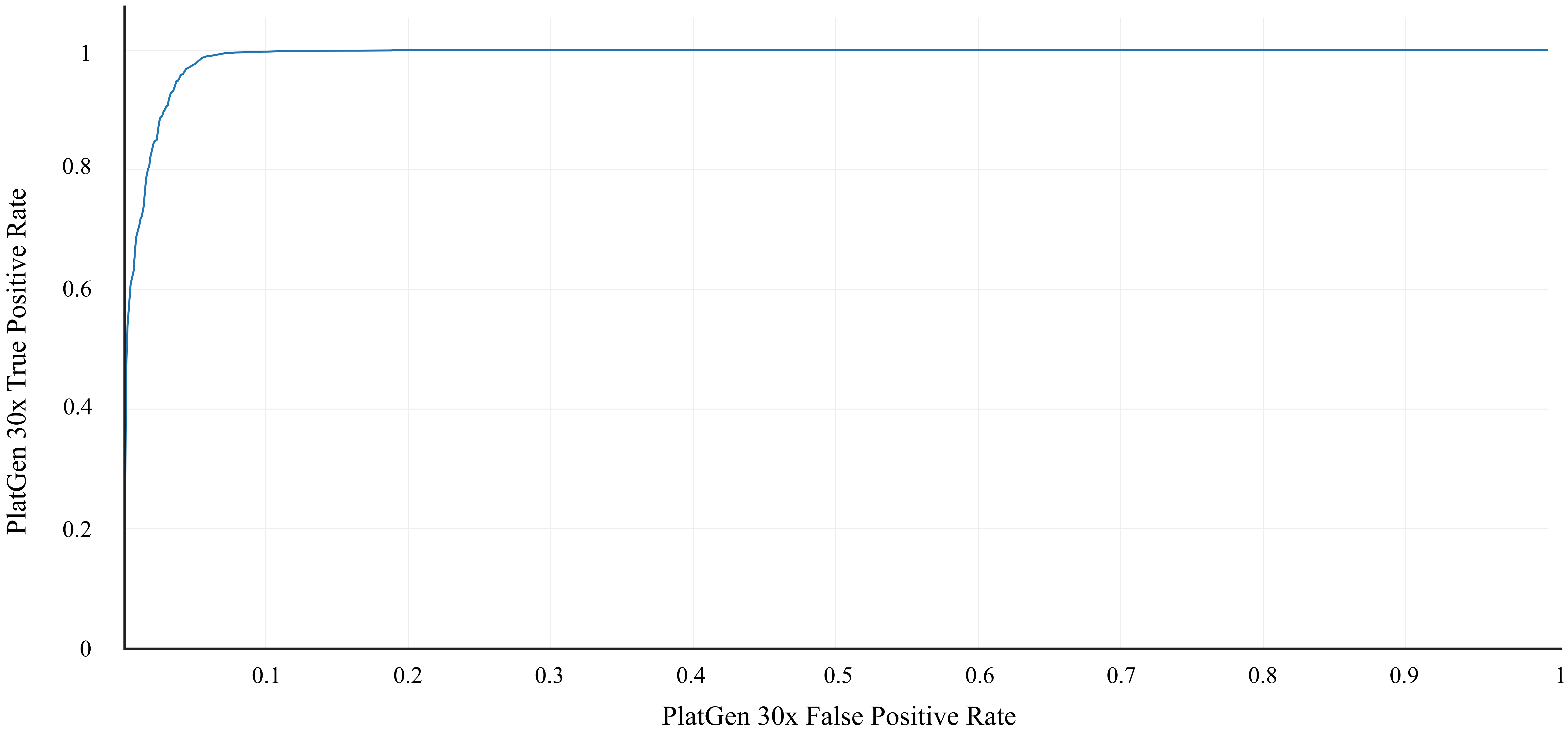

Supplementary figure 7: ROC curves for One-class classification using the L1 Distance, treating the 2306 Random regions (size distribution matching to Personalis) as negatives and the Personalis as positives using the Platinum down-sampling 30x data. See original data at https://plot.ly/165/~parikhhm/.
