# Supplementary material for: svclassify: a method to establish benchmark structural variant calls
Source: BMC Genomics. 2016 Jan 16;17:64. doi: 10.1186/s12864-016-2366-2 (PMC4715349; doi:10.1186/s12864-016-2366-2)
Supplement: Additional file 22: Table S15. — Number of overlapping deletion calls between Personalis and 1000 Genomes deletion calls with different amounts of overlap. (DOC 29 kb) [file 12864_2016_2366_MOESM22_ESM.docx]

**Supplementary table 15**: Number of overlapping deletion calls between Personalis and 1000 Genomes deletion calls with different amounts of overlap.

| **Personalis unique deletion calls** | **1000 Genomes unique deletion calls** | **Overlap** | **# of overlapping deletion calls** |
| --- | --- | --- | --- |
| 2336 | 1825 | 1 bp | 1082 |
| 2336 | 1825 | 10 % | 1082 |
| 2336 | 1825 | 25 % | 1081 |
| 2336 | 1825 | 50 % | 1076 |
| 2336 | 1825 | 75 % | 1070 |
| 2336 | 1825 | 90 % | 1066 |
| 2336 | 1825 | 100 % | 986 |
