# Supplementary material for: svclassify: a method to establish benchmark structural variant calls
Source: BMC Genomics. 2016 Jan 16;17:64. doi: 10.1186/s12864-016-2366-2 (PMC4715349; doi:10.1186/s12864-016-2366-2)
Supplement: Additional file 23: Table S16. — Output format of svclassify. (DOC 30 kb) [file 12864_2016_2366_MOESM23_ESM.docx]

**Supplementary table 16**: Output format of svclassify.

svclassify generates 85 to 180 annotations for each SV from each aligned sequence data, depending on sequencing technology.

*SV_size*: The SV_size gives the size of a structural variant (SV).

*SV_Cat*: The SV_Cat gives the size distribution of a SV as a categorical value (i.e. SV size of < 100 = 0, SV size of >=100 to <1000 = 1, SV size of >=1000 to <10000 = 2, SV size of >=10000 = 3).

Each SV is characterized in five groups (please refer to Figure 1):

(1) Left flanking region (L)

(2) Left middle flanking region (LM)

(3) Middle regions based on SV coordinates (M)

(4) Right middle flanking region (RM)

(5) Right flanking region (R)

*Cov*: The Cov gives the mean of depth of coverage.

*Cov_sd*: The Cov_sd gives the standard deviation of depth of coverage.

*Cov_pro*: The Cov_pro gives the proportion of the SV with depth of coverage less than 5X.

*Insert*: The Insert gives the mean of insert size of paired reads (samtools flags of -f2).

*Insert_sd*: The Insert_sd gives the standard deviation of insert size of paired reads.

*Insert_10_percentile*: The Insert_10_percentile gives the 10^th^ percentile of insert size distribution of paired reads.

*Insert_90_percentile*: The Insert_90_percentile gives the 90^th^ percentile of insert size distribution of paired reads.

*Dis_unmap*: The Dis_unmap gives numbers of the unmapped mate (samtools flags of -f9 -F 1792).

*Dis_map*: The Dis_map gives numbers of the mapped mate in reverse orientation (samtools flags of -f1 -F 1802).

*Dis_all*: The Dis_all gives numbers of the total paired reads (samtools flag of -f2).

*Dis_unmap_ratio*: The Dis_unmap_ratio gives the ratio of numbers of the unmapped mate to numbers of total paired reads.

*Dis_map_ratio*: The Dis_map_ratio gives the ratio of numbers of the mapped mate in reverse orientation to numbers of total paired reads.

*Mapping_q*: The Mapping_q gives the mean of mapping quality of the reads.

*Mapping_q_sd*: The Mapping_q_sd gives the standard deviation of mapping quality of the reads.

*Mapping_pro*: The Mapping_pro gives the proportion of reads with mapping quality of zero.

*Mapping_10_percentile*: The Mapping_10_percentile gives the 10^th^ percentile of mapping quality distribution of the reads.

*Mapping_90_percentile*: The Mapping_90_percentile gives the 90^th^ percentile of mapping quality distribution of the reads.

*Soft*: The Soft gives the mean of soft clipped bases of the reads.

*Soft_sd*: The Soft_sd gives the standard deviation of soft clipped bases of the reads.

*Soft_pro*: The Soft_pro gives the proportion of the reads with soft clipped bases greater than 5.

*Soft_10_percentile*: The Soft_10_percentile gives the 10^th^ percentile of soft clipped bases of the reads distribution.

*Soft_90_percentile*: The Soft_90_percentile gives the 90^th^ percentile of soft clipped bases of the reads distribution.

*Del*: The Del gives the mean of deleted bases of the reads.

*Del_sd*: The Del_sd gives the standard deviation of deleted bases of the reads.

*Del_10_percentile*: The Del_10_percentile gives the 10^th^ percentile of deleted bases of the reads distribution.

*Del_90_percentile*: The Del_90_percentile gives the 90^th^ percentile of deleted bases of the reads distribution.

*Ins*: The Ins gives the mean of inserted bases of the reads.

*Ins_sd*: The Ins_sd gives the standard deviation of inserted bases of the reads.

*Ins_10_percentile*: The Ins_10_percentile gives the 10^th^ percentile of inserted bases of the reads distribution.

*Ins_90_percentile*: The Ins_90_percentile gives the 90^th^ percentile of inserted bases of the reads distribution.

*Diff*: The Diff gives the mean of differences between numbers of inserted and numbers of deleted bases of the reads.

*Diff_sd*: The Diff_sd gives the standard deviation of differences between numbers of inserted and numbers of deleted bases of the reads.

*Diff_10_percentile*: The Diff_10_percentile gives the 10^th^ percentile of differences between numbers of inserted and numbers of deleted bases distribution of the reads.

*Diff_90_percentile*: The Diff_90_percentile gives the 90^th^ percentile of differences between numbers of inserted and numbers of deleted bases distribution of the reads.

*M_Cov_Cat*: The M_Cov_Cat gives the coverage distribution of a SV as a categorical based on user defined input coverage_cutoff value.

*M_Homvar*: The M_Homvar gives the number of homozygous SNP genotype calls inside the SV.

*M_Homvar_SV*: The M_Homvar_SV gives the ratio of number of homozygous SNP genotype calls inside the SV to the size of SV.

*M_Hetvar*: The M_Hetvar gives the number of heterozygous SNP genotype calls inside the SV.

*M_Hetvar_SV*: The M_Hetvar_SV gives the ratio of number of heterozygous SNP genotype calls inside the SV to the size of SV.

*M_GCcontent*: The M_GCcontent gives the percentage of GC content to the size of SV.

*M_Sine_Line_Ltr_SV*: The M_Sine_Line_Ltr_SV gives the percentage of short interspersed nuclear elements (SINE), long interspersed nuclear elements (LINE) and long terminal repeat elements (LTR) identified by RepeatMasker to the size of SV.

*M_Simple_Low_Satellite_SV*: The M_Simple_Low_Satellite_SV gives the percentage of simple, low complexity and satellite repeats identified by RepeatMasker to the size of SV.
