# Supplementary material for: svclassify: a method to establish benchmark structural variant calls
Source: BMC Genomics. 2016 Jan 16;17:64. doi: 10.1186/s12864-016-2366-2 (PMC4715349; doi:10.1186/s12864-016-2366-2)
Supplement: Additional file 24: — Supplementary Information. (DOC 82 kb) [file 12864_2016_2366_MOESM24_ESM.docx]

**Supplementary Information**

1. **Data transform for one- class SVM.**

For a certain annotation, the “right-tail” case means outliers should have positive deviations, the “left-tail” case means outliers should have negative deviations, and the “both-tail” case means that outliers could have either positive or negative deviations. Reference deviations were then calculated for different cases. For the left-tail and right-tail cases,

$$\sigma=\sqrt{\frac{\sum_{i=1}^{n} {(x_{i}-x_{extreme})}^{2}}{n}}$$

σ is the reference deviation, *x_i_* is annotation value of the *i*th site from the random regions. *n* is the number of sites from the random regions (*n* = 4000). *x_extreme_* is either the minimum of *x_i_* for the right-tail case, or the maximum of *x_i_* for left-tail case. For any observation *x* of the same annotation for any SV, the transform *y* is

$$y=2/(1+cosh(|x-x_{extreme}|/\sigma))$$

For both-tail case, we define two reference deviations σ_right_ and σ_left_ for either positive or negative deviations from the median *x_med_* of *x_i_,*

$$\sigma_{right}=\sqrt{\frac{\sum_{x_{i}>x_{med}} {(x_{i}-x_{med})}^{2}}{\sum_{x_{i}>x_{med}} 1}},\sigma_{left}=\sqrt{\frac{\sum_{x_{i}<x_{med}} {(x_{i}-x_{med})}^{2}}{\sum_{x_{i}<x_{med}} 1}}$$

The transform *y* is

$y=2/(1+cosh(|x-x_{med}|/\sigma_{right}))$, if *x* > *x_med_*

$y=2/(1+cosh(|x-x_{med}|/\sigma_{left}))$, if *x* < *x_med_*

*y* = 0, if *x* = *x_med_*

Therefore outliers indicating potential SVs approach 0 in this transform, which is required by the application of one-class SVM. In the transformed metric space, linear classifiers were trained by the one-class SVM (implemented with package e1071 in the Comprehensive R Archive Network) with SVs from the random regions as the training set. The proportion of SVs in the training set identified as outliers (false positive rate) 1-*p* was approximately controlled by a factor ν in the training algorithm defined by the authors. In short, ν ∈ (0,1) defines the ratio of penalty induced by margin size (e.g. distance from origin point to the class boundary with linear kernel) and penalty induced by number of outliers in the training set in the total penalty function for soft margin case. Higher ν allows more training data points to be on the outliers side to maximize the margin. Classifiers at different ν’s were then applied to predict other SV data sets.
